# Clinical Response and Hospital Costs of Therapeutic Drug Monitoring for Vancomycin in Elderly Patients

**DOI:** 10.3390/jpm12020163

**Published:** 2022-01-26

**Authors:** Yun Kim, Soohyun Kim, Jinsook Park, Howard Lee

**Affiliations:** 1 Department of Clinical Pharmacology and Therapeutics, Seoul National University College of Medicine and Hospital, Seoul 03080, Korea; mn07013@snu.ac.kr (Y.K.); iyruts@snu.ac.kr (J.P.); 2 Hanyang Medicine-Engineering-Bio Collaborative & Comprehensive Center for Drug Development (MEBC), Hanyang University, Seoul 04763, Korea; 3 Department of Molecular Medicine and Biopharmaceutical Sciences, Graduate School of Convergence Science and Technology, Seoul National University, Seoul 03080, Korea; soohyunkim5@gmail.com; 4 Center for Convergence Approaches in Drug Development, Graduate School of Convergence Science and Technology, Seoul National University, Seoul 03087, Korea; 5 Advanced Institute of Convergence Technology, Suwon-si 16229, Korea

**Keywords:** vancomycin, antibiotics, therapeutic drug monitoring, pharmacoeconomics, elderly

## Abstract

Cost-effectiveness analysis has been widely used to assess and compare the costs and benefits of a clinical service. The cost-effectiveness of vancomycin therapeutic drug monitoring (TDM) has not been studied in the elderly, who are susceptible to vancomycin-induced adverse effects. This study was performed to evaluate if vancomycin TDM is cost-effective in elderly patients in the Republic of Korea. Using the electronic medical records at a tertiary university hospital, we performed a retrospective observational study to evaluate the cost-effectiveness of vancomycin TDM in 850 elderly patients who underwent vancomycin TDM with an appropriate, recommended dosing regimen and 1094 elderly patients who did not. Cost-effectiveness variables such as clinical outcomes and medical expenses were evaluated using univariate and multivariate analyses. The TDM group spent significantly less than the non-TDM group per patient for total medical expenses (by USD 841.40) and medication expenses (by USD 16.70). However, no significant difference was noted between the TDM and non-TDM groups in clinical outcomes such as microbiological cure, prevention of nephrotoxicity, or reduced mortality, irrespective of admission to the intensive care unit. Vancomycin TDM in elderly patients was associated with economic benefits, but not with better clinical outcomes.

## 1. Introduction

Therapeutic drug monitoring (TDM) has played a significant role in individualized pharmacotherapy as TDM services have become readily available in many hospitals [1,2]. Patients may benefit from TDM because it helps attain therapeutic concentrations or prevents concentrations falling into the toxic range, particularly for drugs that have a narrow therapeutic window such as aminoglycosides, vancomycin, digoxin, and immunosuppressants [2]. However, as concerns about rising health care expenses have increased, the cost-effectiveness of TDM has been also questioned [3].

Cost-effectiveness analysis (CEA) is a tool to analyze and compare the costs and outcomes or effects of a treatment or an intervention in monetary units. CEA has been widely used in health care services to aid decision making in the clinical setting [4]. For CEA to be clinically meaningful, it is important to decide which costs and benefits are to be assessed [5,6]. For example, TDM for aminoglycosides has proven to be cost-effective by decreasing nephrotoxicity and mortality [1,7,8]. Likewise, TDM for conventional antiepileptic drugs including phenobarbital, phenytoin, carbamazepine, primidone, and valproic acid was found to be cost-effective or potentially cost-effective by increasing quality of life [5,6]. However, the cost-effectiveness of TDM for other drugs has not been thoroughly investigated [5,6].

Although vancomycin is one of the most frequently monitored drugs, like aminoglycosides, the cost-effectiveness of vancomycin TDM has only been studied in selected populations such as patients with cancer, those admitted to the intensive care unit or co-medicated with other nephrotoxic drugs including aminoglycosides, amphotericin, and acyclovir [9,10]. In those studies, TDM for vancomycin was cost-effective, i.e., it lowered the incidence of nephrotoxicity and shortened the days of hospitalization [9,10].

However, the cost-effectiveness of vancomycin TDM has not been studied in other patient populations, particularly the elderly, who are susceptible to vancomycin-induced adverse effects, e.g., infusion-related toxicities, nephrotoxicity, and possibly ototoxicity. Older age, higher trough plasma vancomycin concentrations, and longer treatment courses in the elderly can put them at an increased risk for vancomycin toxicities [11,12,13,14]. Because the glomerular filtration rate (GFR) is progressively reduced as a person gets older and vancomycin is primarily excreted through the kidney, the clearance of vancomycin is diminished in elderly patients, leading to higher concentrations [15]. Furthermore, the elderly are more likely to suffer from comorbidities. Therefore, elderly patients tend to take more concomitant medications, which can potentially increase the drug–drug interaction and/or lengthen the treatment duration due to adverse events, thereby they are more vulnerable to vancomycin toxicity.

The objective of this study was to evaluate if vancomycin TDM is cost-effective in elderly patients. To this end, we retrospectively compared the clinical outcomes and medical expenses of elderly patients who underwent vancomycin TDM and those who did not.

## 2. Materials and Methods

### 2.1. Performance of Vancomycin TDM with Pharmacokinetic Consultation

Based on the vancomycin TDM guidelines issued in 2009 [14], an appropriate dosing regimen for vancomycin was recommended by monitoring trough serum vancomycin concentrations. Trough serum vancomycin concentrations were recommended to be at least 10 mg/L to avoid the development of resistant strains or 15–20 mg/L for complicated infections including meningitis, endocarditis, hospital-acquired pneumonia, and osteomyelitis, and improved penetration. A group of clinical pharmacologists, who are medical doctors, clinical pharmacists, or clinical pharmacokineticists performed vancomycin TDM under the supervision of professors at the Department of Clinical Pharmacology and Therapeutics, Seoul National University Hospital by thoroughly reviewing the patients’ medical records such as clinical history, microbial information, dosing regimen with actual history and the results of laboratory tests. In this study, the term TDM was used as the measurement of vancomycin serum concentration along with pharmacokinetic consultation for individual elderly patients. Pharmacokinetic calculations were performed with the ABBOTTBASE^®^ Pharmacokinetic Systems software (version 1.10, Abbott Laboratories, Abbott Park, IL, USA). Using the results from the software, the clinical pharmacologists performed vancomycin TDM and provided the most appropriate dosing and monitoring regimen for the clinical settings.

### 2.2. Study Design and Patients

The clinical data and medical expense information were retrospectively obtained from the electronic medical records in Seoul National University Hospital, Seoul, Republic of Korea, which also contained health insurance reimbursement data. Patients ≥ 65 years of age who received vancomycin for at least for 72 h any time during their hospitalization from January 2009 to December 2013 were eligible for inclusion [16]. Depending on the TDM status and its temporal relationship with vancomycin treatment (VT), patients were divided into two groups: TDM and non-TDM groups. Patients who underwent the initial TDM with the appropriate, recommended dosing regimen within three days of starting VT were classified as the TDM group. In contrast, patients who did not receive TDM at all while they were admitted or those who underwent TDM after three days of VT were assigned to the non-TDM group. If a patient received vancomycin more than one time, only the first treatment was included. However, if the second treatment was not >2 months after the first one, the patient was excluded. Likewise, patients were excluded when the difference in the duration between VT and hospitalization exceeded 30 days. Additionally, patients who had extreme or biologically implausible clinical or laboratory test results were considered as outliers and excluded.

This study was approved by the Institutional Review Board (IRB) of Seoul National University Hospital (IRB No. 1410-042-616), Jongno-gu, Seoul, Republic of Korea. Because this study was based on a retrospective analysis of anonymized electronic medical records, obtaining informed consent was not required by the Institutional Review Board at the Seoul National University Hospital. All procedures were performed in accordance with the recommendations of the Declaration of Helsinki.

### 2.3. Identificaition of Microorganisms and Quantification of Vancomycin Concentration

Blood culture and identification of microorganisms were performed by using BacT/ALERT FA/FN (BioMérieux Inc., Durham, NC, USA). All blood bottles were cultured at 37 °C for 7 days, and identification was performed using an automated system (Microscan WalkAway-96, Siemens Healthcare diagnostics, Deerfield, IL, USA).

Vancomycin serum concentration was measured at the Department of Laboratory Medicine in Seoul National University Hospital as part of the TDM currently being implemented, and was measured by fluorescence polarization immunoassay using TDx assay system (Abbott Laboratories, Chicago, IL, USA). The lowest limit of quantitation in this assay was 0.6 mg/L.

### 2.4. Cost-Effectiveness Variables

We extracted the following information from each patient’s record: sex, age, body weight, height, serum creatinine (Scr), GFR, white blood cell (WBC) count, high sensitivity C-reactive protein (hs-CRP), body temperature, dates and dosing regimens of VT, concomitant antibiotics and antiviral/antifungal agents (whether they are nephrotoxic or not), cultured infectious microorganisms, dates of hospitalization and discharge, dates of admission to and discharge from the intensive care unit (ICU) if applicable, and death or survival during hospitalization. Patients were assigned to the ICU group if they were admitted to the ICU at least once or the non-ICU group.

Using the extracted information, we derived the following continuous clinical cost-effectiveness variables: duration of VT, daily vancomycin dose, length of hospital stay, and duration of fever (up until 5 days after VT). Furthermore, the following binary clinical cost-effectiveness variables were derived: microbiological cure, nephrotoxicity, and mortality. We judged that microbiological cure was achieved if any of the following conditions were met: (1) the patient did not die within 14 days after the end of VT, (2) fever did not continue for more than 5 days after the end of VT, or (3) the microorganism culture was negative for 7 days after the end of VT. Likewise, nephrotoxicity was defined as any of the following conditions: (1) increase in Scr by >0.5 mg/dL from baseline, (2) >50% increase in Scr from baseline, or (3) >50% decrease in the estimated creatinine clearance using the Cockcroft–Gault equation [14,17,18,19,20]. Finally, mortality was documented if it occurred within 14 days after the end of VT.

Additionally, using the health insurance reimbursement data in the electronic medical records, costs for the following medical expenses were calculated: hospitalization, medications, laboratory tests, TDM services, and the total, which might have included other expenses incurred over the period of VT. The medical expenses were converted to US dollars (USD) at the exchange rate of 2017.

### 2.5. Statistical Analysis

CEA was performed separately for the ICU group and non-ICU group. Univariate or unadjusted analyses were conducted first to compare the TDM and non-TDM groups using the two-sample *t*-test or Mann–Whitney U test for continuous variables and the chi-square test for binary variables. Then, multivariate or adjusted analyses, i.e., the analysis of covariance (ANCOVA) and multiple logistic regression analysis were performed for continuous and binary variables, respectively. The covariates for the multivariate analyses included sex, age, body weight, height, baseline values of Scr, GFR, hs-CRP, and WBC, ICU admission (yes or no) or the duration of ICU admission, use of nephrotoxic comedications (yes or no), and the number of nephrotoxic comedications. Nephrotoxic comedications included antimicrobials (aminoglycosides (amikacin, gentamicin, tobramycin, streptomycin), sulfonamides (sulfamethoxazole/trimethoprim), fluroquinolone (ciprofloxacin), polymyxins (colistin, polymycin B)), antiviral agents (acyclovir, foscarnet, antiretroviral drugs), and antifungal agents (amphotericin B) [21,22]. Using the final multiple logistic regression model, the probabilities for developing binary clinical outcomes were predicted. SAS (version 9.4, SAS Institute Inc., Cary, NC, USA) was used for statistical analyses, and a *p*-value of ≤0.05 was considered statistically significant.

## 3. Results

### 3.1. Patients

We initially identified 2303 patients who received vancomycin for at least for 72 h. Of these, 63 patients who received oral vancomycin were excluded. Additionally, 277 patients were excluded because the duration between VT and hospitalization was >30 days. Furthermore, 19 patients were removed because they had one or more extreme or biologically implausible clinical or laboratory test results, and were considered outliers. As a result, a total of 1944 patients were finally included for CEA.

### 3.2. Baseline Demographic and Clinical Characteristics 

Eight-hundred-fifty (43.7%) and 1094 patients (56.3%) were classified into the TDM and the non-TDM groups, respectively. At baseline, the TDM group (mean: 73.7 years and 57.3 kg) was significantly older by 1.1 years and weighed less by 1.6 kg compared to the non-TDM group (mean: 72.6 years and 58.9 kg), whereas the sex distribution (%females) was similar in the TDM (35.5%) and non-TDM (37.8%) groups. In general, the TDM group had significantly worse clinical conditions than the non-TDM group, i.e., lower renal function (mean GFR: 81.3 vs. 83.9 mL/min/1.73 m^2^), higher levels of inflammation (mean hs-CRP: 11.9 vs. 8.9 mg/dL), and more frequent and higher use of nephrotoxic comedications (frequency/no. of use: 23.1%/1.3 vs. 19.3%/1.2). Therefore, all the baseline characteristics that were significantly different between the TDM and non-TDM groups were incorporated as covariates in the multivariate analyses (Table 1).

### 3.3. Univariate Cost-Effectiveness Analysis

The TDM group had significantly worse clinical outcomes than the non-TDM group, i.e., longer duration of VT (mean: 10.8 vs. 9.5 days), longer stay in hospital (mean: 17.4 vs. 16.3 days), more days with fever (mean: 1.7 vs. 1.4 days), lower rate of microbiological cure (61.2% vs. 71.7%), and higher mortality (27.5% vs. 18.2%) (Table 2). In addition, nephrotoxicity was more frequently developed in the TDM group (31.8%) than in the non-TDM group (28.1%) although it was not statistically significant. However, the daily dose of vancomycin (mg/day/kg) was significantly lower in the TDM group (29.8) than in the non-TDM group (31.0). The differences in the clinical outcomes between the TDM and non-TDM groups were generally similar regardless of ICU admission.

The TDM group paid significantly more on average for hospitalization (USD 1234.6 vs. USD 967.8 and laboratory tests (USD 1251.20 vs. USD 1092.80) than the non-TDM group (Table 3). However, the medication expenses and the total medical expenses were significantly lower in the TDM group than in the non-TDM group (USD 62.80 vs. USD 87.10 in medication expenses, *p* = 0.0278; USD 6623.60 vs. USD 8388.10 in total expenses, *p* = 0.007, Table 3). As seen in the clinical outcomes, the differences in the medical expenses between the TDM and non-TDM groups were generally similar irrespective of ICU admission.

### 3.4. Multivariate Cost-Effectiveness Analysis

Unlike the results from the univariate analysis, only the duration of VT was statistically longer by 0.9 of a day in the TDM group compared to the non-TDM group, for the total population (11.7 vs. 10.8 days, *p* = 0.0439, Table 4). The other continuous clinical outcomes, i.e., daily vancomycin dose (29.2 vs. 29.8 mg/day/kg), length of hospital stay (18.5 vs. 17.5 days), and duration of fever (1.7 vs. 1.5 days), were comparable between the TDM and non-TDM groups in total or irrespective of ICU admission.

**Figure 1:**
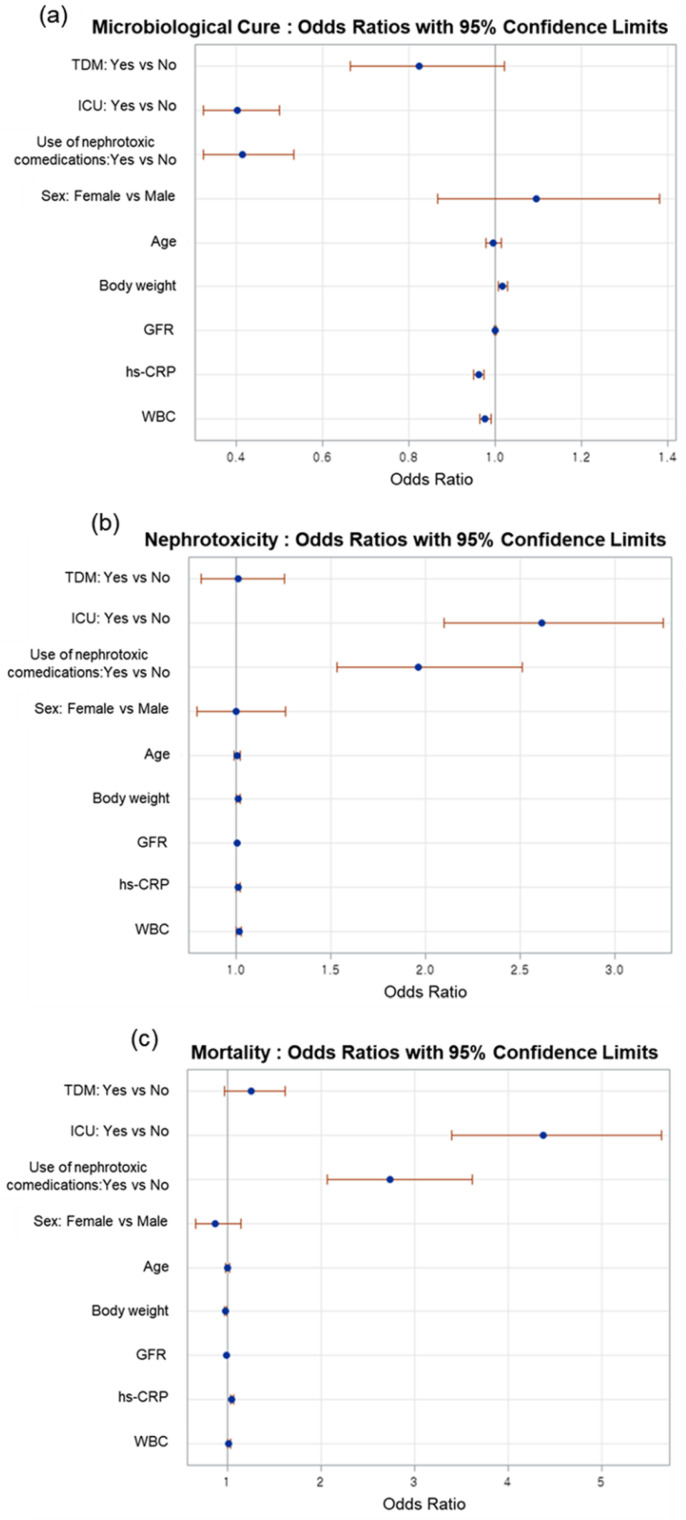
Odds ratios and their 95% confidence limits by TDM and other covariates for (**a**) microbiological cure, (**b**) nephrotoxicity, and (**c**) mortality: multiple logistic regression analysis. Abbreviations: TDM (therapeutic drug monitoring); ICU (intensive care unit); GFR (glomerular filtration rate); hs-CRP (high sensitivity C-reactive protein); WBC (white blood cell).

Furthermore, none of the binary clinical outcomes were significantly different between the TDM and non-TDM groups. For example, all the odds ratios of undergoing vancomycin TDM for microbiological cure, nephrotoxicity, and mortality in the total population were close to 1 and their 95% confidence limits included 1, i.e., 0.824 (95% confidence limits: 0.664–1.022), 1.011 (0.813–1.256), and 1.255 (0.976–1.615), respectively (Figure 1, Table S1). Although the TDM group paid significantly more for hospitalization (USD 1342.70 vs. USD 1155.50) and laboratory tests (USD 1611.50 vs. USD 1505.30) than the non-TDM group, medication (USD 74.70 vs. USD 91.40) and total expenses (USD 8661.40 vs. USD 9502.80) were significantly lower in the TDM group than in the non-TDM group (Table 4).

**Figure 2:**
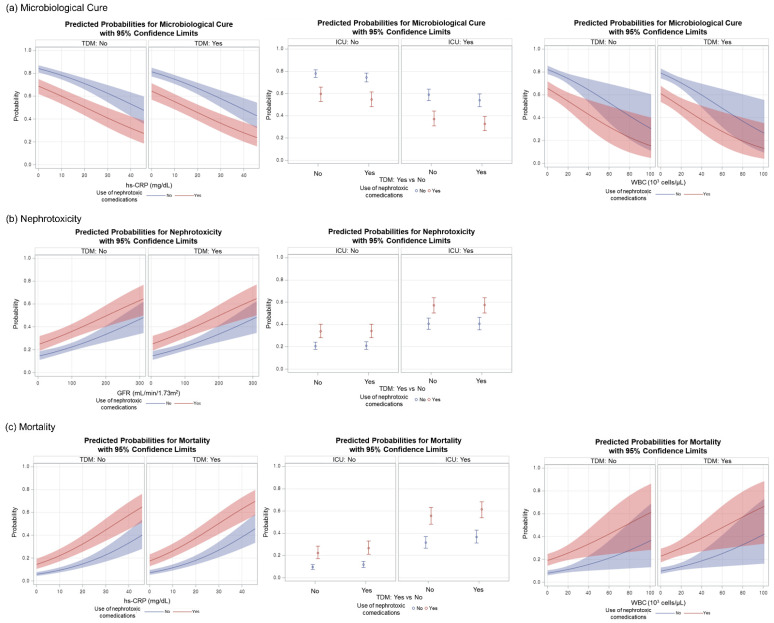
Predicted probability and their 95% confidence limits by TDM and other covariates for (**a**) microbiological cure, (**b**) nephrotoxicity, and (**c**) mortality: multiple logistic regression analysis. Abbreviations: TDM (therapeutic drug monitoring); ICU (intensive care unit); GFR (glomerular filtration rate); hs-CRP (high sensitivity C-reactive protein); WBC (white blood cell).

In both the TDM and non-TDM group, patients with ICU admission, use of nephrotoxic comedications, higher baseline values of hs-CRP and WBC were less likely to experience microbiological cure while they were more likely to die (Figure 2). Patients who used nephrotoxic medications were also predicted to develop nephrotoxicity more frequently, whereas, surprisingly, elderly patients with a higher baseline of GFR had a higher probability of nephrotoxicity (Figure 2).

## 4. Discussion

We showed that vancomycin TDM could be cost-effective in elderly patients in that the total medical expenses and medication expenses were significantly lower in the TDM group than in the non-TDM group. The evidence is that the TDM group spent USD 841.40 (*p* = 0.0062) and USD 16.70 (*p* = 0.0178) less for the total medical and medication expenses, respectively, than the non-TDM group (Table 4). However, the difference in total medical expenses between the two groups became statistically non-significant when analyzed separately for ICU admission (non-ICU, *p* = 0.1068; ICU, *p* = 0.1007, Table 4). Moreover, for other cost-effectiveness variables such as clinical benefits, our data did not support the notion that vancomycin TDM in elderly patients is cost-effective. No significant difference was seen between the TDM and non-TDM groups in the duration of VT, vancomycin dosage, length of hospital stay, or duration of fever regardless of ICU admission (Table 4). Furthermore, mortality and nephrotoxicity were not significantly reduced and the chance of microbiological cure was not significantly increased in the TDM group (Figure 1). What is more, the TDM group spent significantly more on hospitalization and laboratory tests than the non-TDM group (Table 4). These findings were obtained after each cost-effectiveness-related outcome was adjusted by significant patient characteristics. In contrast to the results of univariate analysis, which varied depending on the occurrence of ICU admission across cost-effective variables, multivariate or adjusted analyses generally confirmed that there was no evidence of cost-effectiveness.

Our results were different from those of some previous reports that showed vancomycin TDM was cost-effective by preventing nephrotoxicity in patients with hematologic malignancies, intensive care, and nephrotoxic comedication [9,10]. Another previous study reported that vancomycin TDM was associated with clinical benefits such as decreased vancomycin dosage, shortened duration of VT, and decreased length of hospitalization, although they were not statistically significant [23]. One possible explanation for the discrepancies is that elderly patients with clinically worse conditions underwent more frequent vancomycin TDM services. In our study, elderly patients in the TDM group were clinically worse in general than those in the non-TDM group; they were significantly older and had a higher Scr, lower GFR, higher WBC, and higher CRP at baseline (Table 1). Therefore, we adjusted for the differences in those covariates in our multivariate analyses, but we could still not demonstrate that vancomycin TDM was cost-effective in terms of several clinical outcomes and medical expenses. The other possible explanation is that the elderly patients in our population have received vancomycin TDM services without appropriate interpretation and recommendations [24]. Therefore, vancomycin TDM was not effective enough to reverse the clinical conditions in the elderly in this study. Our findings suggest that the benefit in the TDM group, i.e., lower medication and total medical expenses, may be eradicated by the high expenses in other areas such as hospitalization and laboratory tests and no significant difference in clinical outcomes.

Several covariates were significantly associated with the binary cost-effectiveness variable of vancomycin TDM. First, the use of nephrotoxic comedications significantly increased nephrotoxicity and mortality while it significantly decreased the chance of microbiological cure, regardless of ICU admission (Table S1). Therefore, it is recommended to avoid other nephrotoxic medications as much as possible in elderly patients, particularly those receiving VT [21]. Second, worse clinical outcomes were associated with elderly patients with an increased baseline of hs-CRP, WBC, and GFR (Figure 2 and Table S1). It is rather counterintuitive that the higher the baseline of GFR in elderly patients was, the greater the risk of nephrotoxicity (Figure 2 and Table S1). This unexpected finding suggests that elderly patients with a higher baseline of GFR are more likely to experience a greater decline in renal function [25].

TDM for vancomycin in this study was performed to maintain minimum serum trough concentrations >10 mg/L to avoid the development of resistance according to the previous guidelines [14]. As a recent update, a ratio of the area under the concentration curve to minimum inhibitory concentration (AUC/MIC) of ≥400 (with the MIC driven by broth microdilution (BMD)) is the current recommended target for vancomycin [26]. The minimum trough concentration has been required to be 15 mg/L to achieve a target AUC/MIC of 400 for a pathogen with an MIC of 1 mg/L. Meanwhile, previous studies showed that bacterial susceptibility to vancomycin was significantly shifted over a five-year surveillance period, leading to the question of whether the cut-off point for vancomycin resistance should be lowered [27,28,29]. However, recent international studies indicated that vancomycin MICs have remained unchanged over time, i.e., methicillin-resistant Staphylococcus aureus (MRSA) susceptibility: MIC of ≤1 mg/L for more than 90% of isolates [30,31]. In addition, a global surveillance research showed that no signs of MIC creep over 20 years appeared in 95% of 57,319 isolates [32]. From susceptibility data in South Korea, the oxacillin resistance, which is the standard for the MRSA rate, decreased significantly from 62.2% in 2010 to 46.8% in 2017 [33]. A decrease in resistance to most antibacterial agents was observed, and no S. aureus resistant to vancomycin was found during the investigation period. Vancomycin Resistance Enterococcus showed a resistance rate of 35.0% (48/137) during the period from 28.6% in 2010 to 42.1% in 2017. No antimicrobial resistance of Streptococcus pneumoniae to vancomycin was observed over the recent 10-year period from 2009 to 2018 [34].

Furthermore, various vancomycin susceptibility testing methods may have produced considerable variability in MIC results [26]. The ETEST methodology (bioMérieux) used in this study for the vancomycin susceptibility test, had the lowest agreement with BMD, which could have produced a higher MIC (0.5 to 2 dilutions higher) than BMD [35]. However, the variability in MIC results could have similarly affected both TDM and non-TDM groups. Therefore, we strongly believe that the difference in the cut-offs of bacterial susceptibility by different methods over time could not invalidate the overall conclusion of this study.

According to the performance of vancomycin TDM, it is also important to know whether vancomycin resistance occurs in treated patients. To the best of our knowledge, there was no available clinical research that investigated the association between the performance of TDM and the emergence of vancomycin resistance. Meanwhile, there has been controversy as to whether vancomycin MIC has an impact on the clinical and microbiological outcomes; some reported that a higher vancomycin MIC resulted in a higher probability of treatment failure, but others did not [36,37,38]. A recent retrospective observational study investigating the clinical outcomes of vancomycin TDM in adults reported that the proportion of patients with MIC = 1 mg/L in MRSA infection was significantly higher in the TDM group than in the non-TDM group (57% vs. 24%, *p* = 0.015), while that with MIC ≤ 0.5 mg/L was lower in the TDM group (68% vs. 43%, *p* = 0.114) [39]. As we have shown in this study, this research reported that vancomycin TDM did not result in better microbiological cure, shorter duration of vancomycin treatment, or reduced nephrotoxicity [39]. This study suggests that optimizing vancomycin therapy with appropriate TDM and pharmacokinetic consultation can avoid under-dosing, which contributes to the emergence of vancomycin resistance; however, it does not always lead to clinical effectiveness.

In addition to vancomycin, there are various drug classes that can be used for the treatment of MRSA infections such as lipopeptides (daptomycin), oxazolidinones (linezolid, tedizolid), and fifth-generation cephalosporins (ceftaroline, ceftobiprole) [40]. Clinical use of daptomycin, due to its variability in the pharmacokinetics/pharmacodynamics in populations with special conditions despite receiving standard dose, needs to be supported by the performance of TDM to optimize the dosage and to guarantee therapeutic success, based on the reduced risks of side effects and bacterial resistance [41,42,43]. On the other hand, the current dosing guidelines for linezolid do not include TDM despite its large variability in exposure, and the standard doses may result in inadequate exposure in a significant proportion of patients [44]. Certain populations, such as elderly patients and patients with renal impairment, are more susceptible to the risk of incorrect dosing for linezolid, thereby exposing them to the increased risk of adverse effects including thrombocytopenia and neuropathy. As part of clinical settings, TDM of linezolid with evidence-based thresholds can be a useful tool to maximize therapeutic success and minimize toxicity in most patients [44,45,46]. Ceftaroline and ceftobiprole are fifth-generation cephalosporins with a broad antibacterial spectrum, including potent activity against MRSA [47,48,49]. As for the other cephalosporins, they exhibit high pharmacokinetic variability and concentration-dependent neurotoxicity [50,51,52]. To apply TDM to those drugs in routine practice, methods of quantitative analysis for drugs are being developed [53,54]. However, there is still no research on the cost-effectiveness of TDM for the aforementioned drugs. Although vancomycin TDM was not cost-effective in the elderly in our study, it is necessary to evaluate the cost-effectiveness of other current treatments through additional studies.

This study had several limitations. First, we did not exclude elderly patients with renal impairment at baseline. The inclusion of these patients could have resulted in worse clinical outcomes and an increase in medical expenses. Therefore, vancomycin TDM could appear to be not cost-effective in elderly patients with renal impairment at baseline no matter how effective it was. However, we adjusted for renal function at baseline, such as for GFR and Scr, in the multivariate analyses. Furthermore, it is more important to evaluate the overall cost-effectiveness of vancomycin TDM in elderly patients including those with renal function impairment. Second, misclassification could have happened in this study. For example, we assigned patients to the TDM group only when they underwent the initial TDM within three days of starting VT. Because patients with reduced renal function could have received VT at a dosing interval >24 h, they may have undergone the initial TDM >3 days after the initial VT. Therefore, patients with reduced renal function could have been assigned to the non-TDM group. Nevertheless, our definition of vancomycin TDM was consistent with most vancomycin TDM guidelines that recommend TDM be performed within three days of VT (before the fourth or fifth dose in patients with normal renal function) [16,55,56]. Furthermore, the potential misclassification was weakened in our study by performing multivariate analyses that adjusted for baseline renal functions. Lastly, we did not adjust for the “type of the infection” or “clinical department that prescribed vancomycin” in multivariate analyses. Considering that TDM implementation is commonly affected by the overall policy of a clinical department and by individual clinicians, TDM implementation could have been unevenly distributed among different types of infections and clinical departments. However, interpretation of vancomycin TDM and the recommendation of an appropriate dosing regimen for vancomycin was solely performed by professionally trained practitioners in the Department of Clinical Pharmacology and Therapeutics at a university-affiliated tertiary training hospital, which might minimize the potential bias.

## 5. Conclusions

Vancomycin TDM was associated with economic benefits in elderly patients. However, there was no indication that vancomycin TDM in this population resulted in better clinical outcomes such as microbiological cure, prevention of nephrotoxicity, or reduced mortality. The mere determination of a drug concentration does not ensure the usefulness, effectiveness, or benefit of TDM. Based on the determined drug concentration, TDM must be more adapted to the individual needs (i.e., comedication and baseline characteristics) of the patient to have a chance of improving the therapeutic outcome.

## Figures and Tables

**Table 1:** Baseline demographic and clinical characteristics by ICU admission and TDM group.

Characteristic	Non-ICU (*n* = 1258)				ICU (*n* = 686)				Total (*n* = 1944)				*p*-Value ^1^		
	Non-TDM	*n*	TDM	*n*	Non-TDM	*n*	TDM	*n*	Non-TDM	*n*	TDM	*n*	Non-ICU	ICU	Total
Males/Females/%Females	441/265/37.5	706	338/214/38.8	552	240/148/38.1	388	210/88/29.5	298	681/413/37.8	1094	548/302/35.5	850	0.655	0.0186	0.3136
Age, years	72.3 ± 5.6	706	73.6 ± 6.4	552	73.1 ± 5.9	388	73.8 ± 6.0	298	72.6 ± 5.7	1094	73.7 ± 6.2	850	0.0006	0.0978	0.0002
Body weight, kg	58.1 ± 10.9	704	57.3 ± 11.2	552	60.3 ± 11.2	388	57.5 ± 10.5	298	58.9 ± 11.1	1092	57.3 ± 11.0	850	0.2961	0.001	0.006
Serum creatinine, mg/dL	1.0 ± 0.7	652	1.1 ± 0.9	530	1.2 ± 0.8	370	1.3 ± 0.9	298	1.0 ± 0.7	1022	1.2 ± 0.9	828	0.0063	0.1199	0.0037
Glomerular filtration rate, mL/min/1.73 m^2^	88.6 ± 39.3	652	83.7 ± 45.4	530	75.5 ± 40.4	370	77.1 ± 51.8	298	83.9 ± 40.2	1022	81.3 ± 47.9	828	0.0036	0.2523	0.0058
White blood cell count, 10^3^ cells/μL	9.7 ± 6.1	674	10.7 ± 8.7	537	10.7 ± 7.0	368	13.6 ± 8.4	297	10.0 ± 6.4	1042	11.8 ± 8.7	834	0.05	<0.0001	<0.0001
High sensitivity C-reactive protein, mg/dL	8.0 ± 8.1	620	10.1 ± 7.6	507	10.6 ± 10.0	335	14.9 ± 9.0	294	8.9 ± 8.9	955	11.9 ± 8.4	801	<0.0001	<0.0001	<0.0001
Use of nephrotoxic comedications, %	19.8	706	18.7	552	18.3	388	31.2	298	19.3	1094	23.1	850	0.6017	<0.0001	0.0426
No. of nephrotoxic comedications	1.1 ± 0.4	140	1.3 ± 0.5	103	1.3 ± 0.7	71	1.4 ± 0.7	93	1.2 ± 0.5	211	1.3 ± 0.6	196	0.0053	0.6241	0.00083
Site of infection (%)															
Blood stream	8.6	706	13.6	552	8.2	388	13.1	298	8.5	1094	13.4	850	0.005	0.0391	0.0005
Bone and joint	13.0	706	8.3	552	0.3	388	2.0	298	8.5	1094	6.1	850	0.0081	0.0233	0.0473
Central nervous system	5.9	706	2.4	552	5.2	388	5.0	298	5.7	1094	3.3	850	0.002	0.943	0.0135
Ear, nose and throat	2.0	706	3.8	552	0.8	388	1.7	298	1.6	1094	3.1	850	0.0513	0.274	0.0252
Intra-abdominal	19.8	706	25.9	552	8.2	388	13.8	298	15.7	1094	21.6	850	0.0104	0.0203	0.0008
Reproductive organ	0.8	706	0.9	552	0.3	388	0.7	298	0.6	1094	0.8	850	0.9158	0.416	0.6347
Respiratory	21.5	706	27.0	552	57.2	388	53.7	298	34.2	1094	36.4	850	0.0242	0.3569	0.3209
Skin and soft tissue	9.5	706	4.3	552	1.0	388	0.0	298	6.5	1094	2.8	850	0.0005	0.0788	0.0002
Surgical prophylaxis	15.9	706	9.6	552	17.8	388	9.1	298	16.5	1094	9.4	850	0.0011	0.0011	<0.0001
Urinary tract	2.8	706	4.2	552	1.0	388	1.0	298	2.2	1094	3.1	850	0.1963	0.975	0.232

Abbreviations: TDM (therapeutic drug monitoring); ICU (intensive care unit); *n* (number of patients). Data are shown as mean ± standard deviation. ^1^ The TDM group vs. the non-TDM group. The two-sample *t*-test for continuous variables and the chi-square test for proportions, except for the number of nephrotoxic comedications, for which the Mann–Whitney U test was used.

**Table 2:** Clinical outcomes by ICU admission and TDM group: univariate analysis.

Clinical Outcome	Non-ICU (*n* = 1258)		ICU (*n* = 686)		Total (*n* = 1944)		*p*-Value ^1^		
	Non-TDM (*n* = 706)	TDM (*n* = 552)	Non-TDM (*n* = 388)	TDM (*n* = 298)	Non-TDM (*n* = 1094)	TDM (*n* = 850)	Non-ICU	ICU	Total
Duration of vancomycin treatment, days	10.0 ± 9.7	10.4 ± 7.7	8.5 ± 7.8	11.4 ± 8.2	9.5 ± 9.1	10.8 ± 7.9	<0.0001	<0.0001	<0.0001
Vancomycin dosage, mg/day/kg	33.3 ± 23.1 ^2^	30.5 ± 19.0	26.8 ± 14.1	28.6 ± 29.7	31.0 ± 20.6 ^3^	29.8 ± 23.3	0.0003	0.6104	0.0029
Length of stay in hospital, days	16.3 ± 11.6	17.2 ± 10.2	16.5 ± 10.4	17.9 ± 11.4	16.3 ± 11.2	17.4 ± 10.6	0.0038	0.1804	0.0019
Duration of fever, days	1.9 ± 3.2	2.2 ± 3.4	0.5 ± 1.7	0.7 ± 2.0	1.4 ± 2.9	1.7 ± 3.1	0.0613	0.0087	0.0054
Microbiological cure, *n* (%)	544 (77.1)	399 (72.3)	240 (61.9)	121 (40.6)	784 (71.7)	520 (61.2)	0.0526	<0.0001	<0.0001
Nephrotoxicity, *n* (%)	143 (20.3)	136 (24.6)	164 (42.3)	134 (45.0)	307 (28.1)	270 (31.8)	0.0633	0.4797	0.0763
Mortality, *n* (%)	75 (10.6)	81 (14.7)	124 (32.0)	153 (51.3)	199 (18.2)	234 (27.5)	0.0305	<0.0001	<0.0001

Abbreviations: TDM (therapeutic drug monitoring); ICU (intensive care unit); *n* (number of patients). Data are shown as mean ± standard deviation. ^1^ The TDM group vs. the non-TDM group. The two-sample *t*-test was used for continuous variables, and the chi-square test for proportions. ^2^ *n* = 705; ^3^ *n* = 1093.

**Table 3:** Medical expenses by ICU admission and TDM group: univariate analysis.

Medical Expenses, USD	Non-ICU (*n* = 1258)		ICU (*n* = 686)		Total (*n* = 1944)		*p*-Value ^1^		
	Non-TDM (*n* = 706)	TDM (*n* = 552)	Non-TDM (*n* = 388)	TDM (*n* = 298)	Non-TDM (*n* = 1094)	TDM (*n* = 850)	Non-ICU	ICU	Total
Hospitalization	911.2 ± 1027.1	1143.2 ± 1370.8	1070.8 ± 959.8	1403.8 ± 948.0	967.8 ± 1006.2	1234.6 ± 1244.8	<0.0001	<0.0001	<0.0001
Medications	84.3 ± 156.0	60.0 ± 85.4	92.1 ± 113.8	68.0 ± 94.2	87.1 ± 145.3	62.8 ± 88.6	0.7014	0.0024	0.0278
Laboratory tests	690.4 ± 752.0	748.3 ± 643.5	1825.1 ± 1538.1	2182.8 ± 1461.0	1092.8 ± 1223.6	1251.2 ± 1218.4	0.0005	<0.0001	0.0003
TDM Service	-	89.1 ± 68.1	-	102.0 ± 75.3	-	93.6 ± 71.0	-	-	-
Total ^2^	5048.6 ± 5944.1	4121.7 ± 3933.9	14,464 ± 10,608	11,258 ± 10,015	8388.1 ± 9107.2	6623.6 ± 7532.4	0.5394	<0.0001	0.0070

Abbreviations: TDM (therapeutic drug monitoring); ICU (intensive care unit); *n* (number of patients). Data are shown as mean ± standard deviation. ^1^ The TDM group vs. the non-TDM group. The two-sample *t*-test was used. ^2^ The total could include other expenses incurred during hospitalization than those listed.

**Table 4:** Continuous clinical outcomes and medical expenses by ICU admission and TDM group: multivariate analysis.

Variable	Non-ICU (*n* = 1101)		ICU (*n* = 628)		Total (*n* = 1729)		ANCOVA		
							*p*-Value ^1^		
	Non-TDM (*n* = 602)	TDM (*n* = 499)	Non-TDM (*n* = 334)	TDM (*n* = 294)	Non-TDM (*n* = 936)	TDM (*n* = 793)	Non-ICU	ICU	Total
Duration of vancomycin treatment, days	11.0 (10.2–11.8)	11.4 (10.5–12.3)	10.5 (9.5–11.5)	11.8 (10.8–12.8)	10.8 (10.2–11.4)	11.7 (11.0–12.3)	0.4928	0.0522	0.0439
Vancomycin dosage, mg/day/kg	31.5 (29.6–33.4)	29.8 (27.7–31.8)	27.5 (24.9–30.1)	28.7 (26.0–31.5)	29.8 (28.3–31.4)	29.2 (27.5–30.8)	0.1650	0.4846	0.5169
Length of stay in hospital, days	17.6 (16.6–18.6)	18.6 (17.5–19.8)	17.1 (15.8–18.5)	18.3 (16.9–19.7)	17.5 (16.7–18.3)	18.5 (17.7–19.4)	0.1243	0.2097	0.0531
Duration of fever, days	2.4 (2.1–2.7)	2.6 (2.2–2.9)	0.7 (0.4–0.9)	0.8 (0.6–1.1)	1.5 (1.3–1.8)	1.7 (1.4–1.9)	0.3832	0.3760	0.3897
Medical expenses, USD									
Hospitalization	1026.3 (914.4–1138.3)	1214.0 (1092.5–1335.4)	1320.0 (1204.5–1435.5)	1456.4 (1336.6–1576.2)	1155.5 (1072.2–1238.8)	1342.7 (1254.1–1431.3)	0.0109	0.0832	0.0006
Medications	84.8 (72.4–97.2)	70.0 (56.6–83.5)	97.4 (84.3–110.4)	78.9 (65.4–92.4)	91.4 (82.1–100.6)	74.7 (64.8–84.5)	0.0697	0.0383	0.0062
Laboratory tests	803.1 (740.3–865.8)	862.6 (794.5–930.7)	2240.2 (2059.2–2421.2)	2381.8 (2194.1–2569.6)	1505.3 (1426.8–1583.8)	1611.5 (1527.9–1695.0)	0.1493	0.2509	0.0399
Total ^2^	5583.5 (4649.8–5517.3)	4623.2 (4152.4–5093.9)	14,112 (12,865–15,359)	12,716 (11,422–14,010)	9502.8 (8963.1–10,043)	8661.4 (8087.2–9235.6)	0.1068	0.1007	0.0178

Abbreviations: TDM (therapeutic drug monitoring); ICU (intensive care unit); *n* (number of patients); ANCOVA (analysis of covariance). The covariates included sex, age, body weight, the baseline values of GFR, hs-CRP, and WBC count, ICU admission a week before vancomycin treatment until a week after vancomycin treatment, and use of nephrotoxic comedications. Data are shown as least squares means (95% confidence limits) derived by an analysis of the covariance model. ^1^ The TDM group vs. the non-TDM group; ^2^ The total could include other expenses incurred during hospitalization than those listed.

## Data Availability

The datasets used and/or analyzed during the current study are available from the corresponding author on reasonable request.

## Supplementary Materials

The following are available online at https://www.mdpi.com/article/10.3390/jpm12020163/s1, Table S1: Summary of odds ratios for clinical outcomes by ICU admission, TDM group, and other covariates.

## References

[1] Bond C.A., Raehl C.L. (2005). Clinical and economic outcomes of pharmacist-managed aminoglycoside or vancomycin therapy. Am. J. Health Syst. Pharm..

[2] Ali A.S., Abdel-Rhaman M.S., Osman O.H. (2013). Basic Principles of Therapeutic Drug Monitoring. J. Appl. Biopharm. Pharmacokinet..

[3] Kang J.-S., Lee M.-H. (2009). Overview of therapeutic drug monitoring. Korean J. Intern. Med..

[4] Russell L. (1996). The Role of Cost-effectiveness Analysis in Health and Medicine. J. Am. Med. Assoc..

[5] Haji E.O., Mann K., Dragicevic A., Müller M.J., Boland K., Rao M.-L., Fric M., Laux G., Hiemke C. (2013). Potential cost-effectiveness of therapeutic drug monitoring for depressed patients treated with citalopram. Ther. Drug Monit..

[6] Touw D.J., Neef C., Thomson A.H., Vinks A.A. (2005). Cost-effectiveness of therapeutic drug monitoring: A systematic review. Ther. Drug Monit..

[7] Eisenberg J.M., Koffer H., Glick H.A., Connell M.L., Loss L.E., Talbot G.H., Shusterman N.H., Strom B.L. (1987). What is the cost of nephrotoxicity associated with aminoglycosides?. Ann. Intern. Med..

[8] Streetman D.S., Nafziger A.N., Destache C.J., Bertino J.S. (2001). Individualized Pharmacokinetic Monitoring Results in Less Aminoglycoside-Associated Nephrotoxicity and Fewer Associated Costs. Pharmacother. J. Hum. Pharmacol. Drug Ther..

[9] Darko W., Medicis J.J., Smith A., Guharoy R., Lehmann D.E. (2003). Mississippi mud no more: Cost-effectiveness of pharmacokinetic dosage adjustment of vancomycin to prevent nephrotoxicity. Pharmacotherapy.

[10] Fernandez de Gatta M.D., Calvo M.V., Hernandez J.M., Caballero D., San Miguel J.F., Dominguez-Gil A. (1996). Cost-effectiveness analysis of serum vancomycin concentration monitoring in patients with hematologic malignancies. Clin. Pharmacol. Ther..

[11] Rybak M.J., Albrecht L.M., Boike S.C., Chandrasekar P.H. (1990). Nephrotoxicity of vancomycin, alone and with an aminoglycoside. J. Antimicrob. Chemother..

[12] Ye Z.K., Tang H.L., Zhai S.D. (2013). Benefits of therapeutic drug monitoring of vancomycin: A systematic review and meta-analysis. PLoS ONE.

[13] Farber B.F., Moellering R.C. (1983). Retrospective study of the toxicity of preparations of vancomycin from 1974 to 1981. Antimicrob Agents Chemother.

[14] Rybak M., Lomaestro B., Rotschafer J.C., Moellering R., Craig W., Billeter M., Dalovisio J.R., Levine D.P. (2009). Therapeutic monitoring of vancomycin in adult patients: A consensus review of the American Society of Health-System Pharmacists, the Infectious Diseases Society of America, and the Society of Infectious Diseases Pharmacists. Am. J. Health Syst. Pharm..

[15] Guay D.R.P., Vance-Bryan K., Gilliland S., Rodvold K., Rotschafer J. (1993). Comparison of vancomycin pharmacokinetics in hospitalized elderly and young patients using a Bayesian forecaster. J. Clin. Pharmacol..

[16] Matsumoto K., Takesue Y., Ohmagari N., Mochizuki T., Mikamo H., Seki M., Takakura S., Tokimatsu I., Takahashi Y., Kasahara K. (2013). Practice guidelines for therapeutic drug monitoring of vancomycin: A consensus review of the Japanese Society of Chemotherapy and the Japanese Society of Therapeutic Drug Monitoring. J. Infect. Chemother..

[17] Hidayat L.K., Hsu D.I., Quist R., Shriner K.A., Wong-Beringer A. (2006). High-dose vancomycin therapy for methicillin-resistant Staphylococcus aureus infections: Efficacy and toxicity. Arch. Intern. Med..

[18] Jeffres M.N., Isakow W., Doherty J.A., Micek S.T., Kollef M.H. (2007). A retrospective analysis of possible renal toxicity associated with vancomycin in patients with health care-associated methicillin-resistant Staphylococcus aureus pneumonia. Clin. Ther..

[19] Lodise T.P., Lomaestro B., Graves J., Drusano G.L. (2008). Larger vancomycin doses (at least four grams per day) are associated with an increased incidence of nephrotoxicity. Antimicrob. Agents Chemother..

[20] Han H.K., An H., Shin K.H., Shin D., Lee S.H., Kim J.H., Cho S.H., Kang H.R., Jang I.J., Yu K.S. (2014). Trough concentration over 12.1 mg/L is a major risk factor of vancomycin-related nephrotoxicity in patients with therapeutic drug monitoring. Ther. Drug Monit..

[21] Naughton C.A. (2008). Drug-induced nephrotoxicity. Am. Fam. Physician.

[22] Pazhayattil G.S., Shirali A.C. (2014). Drug-induced impairment of renal function. Int. J. Nephrol. Renov. Dis..

[23] Welty T.E., Copa A.K. (1994). Impact of vancomycin therapeutic drug monitoring on patient care. Ann. Pharm..

[24] Suryadevara M., Steidl K.E., Probst L.A., Shaw J. (2012). Inappropriate vancomycin therapeutic drug monitoring in hospitalized pediatric patients increases pediatric trauma and hospital costs. J. Pediatr. Pharmacol. Ther..

[25] Loh J.M., Tran A.L., Ji L., Groshen S., Daneshmand S., Schuckman A., Quinn D.I., Dorff T.B. (2018). Baseline Glomerular Filtration Rate and Cisplatin- Induced Renal Toxicity in Urothelial Cancer Patients. Clin. Genitourin Cancer.

[26] Rybak M.J., Le J., Lodise T.P., Levine D.P., Bradley J.S., Liu C., Mueller B.A., Pai M.P., Wong-Beringer A., Rotschafer J.C. (2020). Therapeutic monitoring of vancomycin for serious methicillin-resistant Staphylococcus aureus infections: A revised consensus guideline and review by the American Society of Health-System Pharmacists, the Infectious Diseases Society of America, the Pediatric Infectious Diseases Society, and the Society of Infectious Diseases Pharmacists. Am. J. Health Syst. Pharm..

[27] Rhee K.Y., Gardiner D.F., Charles M. (2005). Decreasing in vitro susceptibility of clinical Staphylococcus aureus isolates to vancomycin at the New York Hospital: Quantitative testing redux. Clin Infect. Dis..

[28] Wang G., Hindler J.F., Ward K.W., Bruckner D.A. (2006). Increased vancomycin MICs for Staphylococcus aureus clinical isolates from a university hospital during a 5-year period. J. Clin. Microbiol..

[29] Steinkraus G., White R., Friedrich L. (2007). Vancomycin MIC creep in non-vancomycin-intermediate Staphylococcus aureus (VISA), vancomycin-susceptible clinical methicillin-resistant S. aureus (MRSA) blood isolates from 2001–2005. J. Antimicrob. Chemother..

[30] Diaz R., Afreixo V., Ramalheira E., Rodrigues C., Gago B. (2018). Evaluation of vancomycin MIC creep in methicillin-resistant Staphylococcus aureus infections-a systematic review and meta-analysis. Clin. Microbiol. Infect..

[31] Pfaller M.A., Sader H.S., Flamm R.K., Castanheira M., Smart J.I., Mendes R.E. (2017). In Vitro Activity of Telavancin Against Clinically Important Gram-Positive Pathogens from 69 U.S. Medical Centers (2015): Potency Analysis by U.S. Census Divisions. Microb. Drug Resist..

[32] Diekema D.J., Pfaller M.A., Shortridge D., Zervos M., Jones R.N. (2019). Twenty-Year Trends in Antimicrobial Susceptibilities Among Staphylococcus aureus From the SENTRY Antimicrobial Surveillance Program. Open Forum. Infect. Dis..

[33] Kim J.S., Gong S.Y., Kim J.W., Rheem I., Kim G.Y. (2019). Antimicrobial Susceptibility Patterns of Microorganisms Isolated from Blood Culture during the Last 8 Years: 2010∼2017. Korean J. Clin Lab. Sci..

[34] Oh H., Heo S.T., Kim M., Kim Y.R., Yoo J.R. (2021). Antimicrobial Susceptibility Trends of Streptococcus pneumoniae by Age Groups Over Recent 10 Years in a Single Hospital in South Korea. Yonsei Med. J..

[35] Hsu D.I., Hidayat L.K., Quist R., Hindler J., Karlsson A., Yusof A., Wong-Beringer A. (2008). Comparison of method-specific vancomycin minimum inhibitory concentration values and their predictability for treatment outcome of meticillin-resistant Staphylococcus aureus (MRSA) infections. Int. J. Antimicrob Agents.

[36] van Hal S.J., Lodise T.P., Paterson D.L. (2012). The clinical significance of vancomycin minimum inhibitory concentration in Staphylococcus aureus infections: A systematic review and meta-analysis. Clin. Infect. Dis..

[37] Peleg A.Y., Monga D., Pillai S., Mylonakis E., Moellering R.C., Eliopoulos G.M. (2009). Reduced susceptibility to vancomycin influences pathogenicity in Staphylococcus aureus infection. J. Infect. Dis..

[38] Yoo R.N., Kim S.H., Lee J. (2017). Impact of Initial Vancomycin Trough Concentration on Clinical and Microbiological Outcomes of Methicillin-Resistant Staphylococcus aureus Bacteremia in Children. J. Korean Med. Sci..

[39] Kim S.M., Lee H.S., Hwang N.Y., Kim K., Park H.D., Lee S.Y. (2021). Individualized Vancomycin Dosing with Therapeutic Drug Monitoring and Pharmacokinetic Consultation Service: A Large-Scale Retrospective Observational Study. Drug Des. Devel. Ther..

[40] Gajdacs M. (2019). The Continuing Threat of Methicillin-Resistant Staphylococcus aureus. Antibiotics.

[41] Osorio C., Garzon L., Jaimes D., Silva E., Bustos R.H. (2021). Impact on Antibiotic Resistance, Therapeutic Success, and Control of Side Effects in Therapeutic Drug Monitoring (TDM) of Daptomycin: A Scoping Review. Antibiotics.

[42] Totoli E.G., Garg S., Salgado H.R. (2015). Daptomycin: Physicochemical, Analytical, and Pharmacological Properties. Ther. Drug Monit..

[43] Galar A., Munoz P., Valerio M., Cercenado E., Garcia-Gonzalez X., Burillo A., Sanchez-Somolinos M., Juarez M., Verde E., Bouza E. (2019). Current use of daptomycin and systematic therapeutic drug monitoring: Clinical experience in a tertiary care institution. Int. J. Antimicrob. Agents.

[44] Rao G.G., Konicki R., Cattaneo D., Alffenaar J.W., Marriott D.J.E., Neely M., Committee I.A.S. (2020). Therapeutic Drug Monitoring Can Improve Linezolid Dosing Regimens in Current Clinical Practice: A Review of Linezolid Pharmacokinetics and Pharmacodynamics. Ther. Drug Monit..

[45] Pea F., Cojutti P.G., Baraldo M. (2017). A 10-Year Experience of Therapeutic Drug Monitoring (TDM) of Linezolid in a Hospital-wide Population of Patients Receiving Conventional Dosing: Is there Enough Evidence for Suggesting TDM in the Majority of Patients?. Basic Clin. Pharm. Toxicol..

[46] Falcone M., Russo A., Cassetta M.I., Lappa A., Tritapepe L., d’Ettorre G., Fallani S., Novelli A., Venditti M. (2013). Variability of pharmacokinetic parameters in patients receiving different dosages of daptomycin: Is therapeutic drug monitoring necessary?. J. Infect. Chemother.

[47] Urban E., Stone G.G. (2019). Impact of EUCAST ceftaroline breakpoint change on the susceptibility of methicillin-resistant Staphylococcus aureus isolates collected from patients with complicated skin and soft-tissue infections. Clin. Microbiol. Infect..

[48] Livermore D.M., Mushtaq S., Warner M., James D., Kearns A., Woodford N. (2015). Pathogens of skin and skin-structure infections in the UK and their susceptibility to antibiotics, including ceftaroline. J. Antimicrob. Chemother..

[49] Duplessis C., Crum-Cianflone N.F. (2011). Ceftaroline: A New Cephalosporin with Activity against Methicillin-Resistant Staphylococcus aureus (MRSA). Clin Med. Rev. Ther..

[50] Torres A., Mouton J.W., Pea F. (2016). Pharmacokinetics and Dosing of Ceftobiprole Medocaril for the Treatment of Hospital- and Community-Acquired Pneumonia in Different Patient Populations. Clin. Pharm..

[51] Kiang T.K., Wilby K.J., Ensom M.H. (2015). A critical review on the clinical pharmacokinetics, pharmacodynamics, and clinical trials of ceftaroline. Clin. Pharm..

[52] Cies J.J., Moore W.S., Enache A., Chopra A. (2018). Ceftaroline for Suspected or Confirmed Invasive Methicillin-Resistant Staphylococcus aureus: A Pharmacokinetic Case Series. Pediatr. Crit. Care Med..

[53] Llopis B., Bleibtreu A., Schlemmer D., Robidou P., Paccoud O., Tissot N., Noe G., Junot H., Luyt C.E., Funck-Brentano C. (2021). Simple and accurate quantitative analysis of cefiderocol and ceftobiprole in human plasma using liquid chromatography-isotope dilution tandem mass spectrometry: Interest for their therapeutic drug monitoring and pharmacokinetic studies. Clin Chem. Lab. Med..

[54] Lima B., Bodeau S., Quinton M.C., Leven C., Lemaire-Hurtel A.S., Bennis Y. (2019). Validation and Application of an HPLC-DAD Method for Routine Therapeutic Drug Monitoring of Ceftobiprole. Antimicrob. Agents Chemother.

[55] Ye Z.K., Li C., Zhai S.D. (2014). Guidelines for therapeutic drug monitoring of vancomycin: A systematic review. PLoS ONE.

[56] Rybak M.J., Lomaestro B.M., Rotschafer J.C., Moellering R.C., Craig W.A., Billeter M., Dalovisio J.R., Levine D.P. (2009). Vancomycin therapeutic guidelines: A summary of consensus recommendations from the infectious diseases Society of America, the American Society of Health-System Pharmacists, and the Society of Infectious Diseases Pharmacists. Clin. Infect. Dis..

