# Supplementary material for: Clinical Response and Hospital Costs of Therapeutic Drug Monitoring for Vancomycin in Elderly Patients
Source: J Pers Med. 2022 Jan 26;12(2):163. doi: 10.3390/jpm12020163 (PMC8875716; doi:10.3390/jpm12020163)
Supplement: Supplementary file 1 [file jpm-12-00163-s001.zip › jpm-1473068-supplementary.pdf]

Table S1. Summary of odds ratios for clinical outcomes by ICU admission, TDM group, and other covariates

Clinical outcome	ICU Admission	Covariate	Odds ratio	95% CL	p-value
Microbiological cure	Non-ICU	TDM	0.916	0.693 - 1.210	0.5356
		hs-CRP	0.959	0.943 - 0.976	<0.0001
		Use of nephrotoxic comedications	0.621	0.444 - 0.870	0.0056
		GFR	0.996	0.993 - 0.999	0.0197
		WBC	0.981	0.964 - 0.997	0.0247
	ICU	TDM	0.725	0.507 - 1.037	0.0787
		Use of nephrotoxic comedications	0.208	0.135 - 0.320	<0.0001
		hs-CRP	0.967	0.950 - 0.986	0.0005
		GFR	1.006	1.002 - 1.010	0.0033
		WBC	0.972	0.949 - 0.995	0.0162
		Body weight	1.019	1.002 - 1.037	0.0282
	Total	TDM	0.824	0.664 - 1.022	0.0782
		ICU admission	0.402	0.322 - 0.500	<0.0001
		Use of nephrotoxic comedications	0.415	0.323 - 0.533	<0.0001
		hs-CRP	0.962	0.950 - 0.974	<0.0001
		WBC	0.977	0.964 - 0.991	0.0009
		Body weight	1.017	1.006 - 1.028	0.0018
Nephrotoxicity	Non-ICU	TDM	1.160	0.869 - 1.547	0.3136
		GFR	1.007	1.004 - 1.011	<0.0001
		Use of nephrotoxic comedications	1.762	1.253 - 2.477	0.0011
		WBC	1.020	1.002 - 1.038	0.0283
	ICU	TDM	0.859	0.612 - 1.205	0.3776
		Use of nephrotoxic comedications	2.357	1.619 - 3.433	<0.0001
		GFR	1.004	1.000 - 1.007	0.0498
	Total	TDM	1.011	0.813 - 1.256	0.9241
		ICU admission	2.612	2.095 - 3.257	<0.0001
		Use of nephrotoxic comedications	1.959	1.529 - 2.511	<0.0001
		GFR	1.006	1.003 - 1.008	<0.0001
Mortality	Non-ICU	TDM	1.181	0.818 - 1.705	0.3751
		Use of nephrotoxic comedications	1.942	1.274 - 2.961	0.0020
		hs-CRP	1.069	1.047 - 1.091	<0.0001
	ICU	TDM	1.335	0.935 - 1.905	0.1116
		Use of nephrotoxic comedications	4.017	2.689 - 5.999	<0.0001
		hs-CRP	1.042	1.023 - 1.061	<0.0001
	Total	TDM	1.255	0.976 - 1.615	0.0767
		ICU admission	4.383	3.405 - 5.643	<0.0001
		Use of nephrotoxic comedications	2.737	2.072 - 3.616	<0.0001

hs-CRP	1.054	1.039 - 1.068	<0.0001
Body weight	0.984	0.971 - 0.996	0.0101
WBC	1.019	1.004 - 1.034	0.0115

Abbreviations: TDM (therapeutic drug monitoring); ICU (intensive care unit); hs-CRP (high sensitivity C-reactive protein); GFR (glomerular filtration rate); WBC (white blood cell); CL, confidence limits

The covariates included sex, age, body weight, the baseline values of GFR, hs-CRP, and WBC count, ICU admission a week before vancomycin treatment until a week after vancomycin treatment, and use of nephrotoxic comedications
